# Advancements in Artificial Intelligence and Machine Learning for Occupational Risk Prevention: A Systematic Review on Predictive Risk Modeling and Prevention Strategies

**DOI:** 10.3390/s25175419

**Published:** 2025-09-02

**Authors:** Pablo Armenteros-Cosme, Marcos Arias-González, Sergio Alonso-Rollán, Sergio Márquez-Sánchez, Albano Carrera

**Affiliations:** BISITE Research Group, University of Salamanca, C. Espejo, 2, 37007 Salamanca, Spain; marcosusal@usal.es (M.A.-G.); sergio.alro@usal.es (S.A.-R.); smarquez@usal.es (S.M.-S.)

**Keywords:** artificial intelligence, machine learning, risk prevention, occupational safety, systematic literature mapping

## Abstract

**Background:** Occupational risk prevention is a critical discipline for ensuring safe working conditions and minimizing accidents and occupational diseases. With the rise of artificial intelligence (AI) and machine learning (ML), these approaches are increasingly utilized for predicting and preventing workplace hazards. This systematic review aims to identify, evaluate, and synthesize existing literature on the use of AI algorithms for detecting and predicting hazardous environments and occupational risks in the workplace, focusing on predictive modeling and prevention strategies. **Methods:** A systematic literature review was conducted following the PRISMA 2020 protocol, with minor adaptations to include conference proceedings and technical reports due to the topic’s emerging and multidisciplinary nature. Searches were performed in IEEE Digital Library, PubMed, Scopus, and Web of Science, with the last search conducted on 1 August 2024. Only peer-reviewed articles published from 2019 onwards and written in English were included. Systematic literature reviews were explicitly excluded. The screening process involved duplicate removal (reducing 209 initial documents to 183 unique ones), a preliminary screening based on titles, abstracts, and keywords (further reducing to 92 articles), and a detailed full-text review. During the full-text review, study quality was assessed using six quality assessment (QA) questions, where articles receiving a total score below 4.5 or 0 in any QA question were excluded. This rigorous process resulted in the selection of 61 relevant articles for quantitative and qualitative analysis. **Results:** The analysis revealed a growing interest in the field, with a clear upward trend in publications from 2021 to 2023, and a continuation of growth into 2024. The most significant contributions originated from countries such as China, South Korea, and India. Applications primarily focused on high-risk sectors, notably construction, mining, and manufacturing. The most common approach involved the use of visual data captured by cameras, which constituted over 40% of the reviewed studies, processed using deep learning (DL) models, particularly Convolutional Neural Networks (CNNs) and You Only Look Once (YOLO). **Conclusions:** The study highlights current limitations, including an over-reliance on visual data (especially challenging in low-visibility environments) and a lack of methodological standardization for AI-based risk detection systems. Future research should emphasize the integration of multimodal data (visual, environmental, physiological) and the development of interpretable AI models (XAI) to enhance accuracy, transparency, and trust in hazard detection systems. Addressing long-term societal implications, such as privacy and potential worker displacement, necessitates transparent data policies and robust regulatory frameworks.

## 1. Introduction

Occupational risk prevention is a fundamental pillar in workplace safety management across various environments [1]. This proactive approach aims to anticipate and address hazards before incidents occur, thereby safeguarding workers’ health [2]. Exposure to harmful environments and risky behaviors by workers can lead to accidents and occupational diseases, with significant impacts on both employees and organizations [3]. Indeed, the ILO estimates that around 2.9 million work-related fatalities occur globally each year—most due to chronic illnesses—underscoring the urgency of better prevention strategies [2]. Simultaneously, the rise of Industry 4.0 has transformed workplaces: interconnected IoT sensors, wearable devices, and cameras now stream real-time data on worker behavior and environmental conditions [4]. These approaches enable the analysis of vast datasets generated by diverse devices—such as environmental sensors, cameras, wearables, and micro-electromechanical systems (MEMS)—helping to uncover patterns and develop predictive models that enhance decision-making in safety management [5].

Beyond data collection, artificial intelligence (AI) methods are increasingly embedded into safety systems, enabling continuous monitoring and early interventions. Recent studies emphasize that the integration of wearable and IoT-enabled devices provides continuous ergonomic and physiological monitoring, which can significantly reduce musculoskeletal risks [6] and improve worker efficiency [7]. In parallel, vision-based AI systems have shown strong potential for automating the detection of unsafe behaviors and compliance with personal protective equipment (PPE), offering a scalable and proactive approach to risk management in hazardous environments [8].

Recent advances in computational sciences have facilitated the creation of more precise, context-specific tools tailored to the unique demands of high-risk industries, such as mining [9,10,11], manufacturing [12] and construction [13,14,15,16]. In construction, for instance, computer vision (CV) algorithms combined with deep learning (DL) models have been successfully applied to video streams for real-time detection of fall risks and verification of PPE usage, demonstrating measurable improvements in safety monitoring [17]. These sector-specific applications illustrate that AI-driven approaches must be adapted to the distinct hazards, workflows, and organizational structures of each industry, reinforcing the need for systematic evaluation across contexts.

This paper presents a systematic review of the literature on the use of AI technologies for predicting and preventing occupational hazards. The objective is to identify key trends, methodological approaches, and challenges, with a particular focus on how these technologies are applied across industries and their impact on workplace safety. Additionally, the paper addresses current limitations and discusses potential future developments in building more effective, tailored systems for risk prevention. The structure of the article is organized as follows: Section 2 presents the methodology, describing the research design; Section 3 details the review process, including the criteria and procedures for selecting studies; Section 4 reports the results obtained from the analysis; Section 5 provides a detailed analysis of patterns and implications; Section 6 is dedicated to the discussion of the outcomes in a broader context; and Section 7 outlines future work, highlighting possible directions for advancing research in this area.

## 2. Methodology

Across academic and professional settings, systematic literature reviews play a crucial role in ensuring the validity, methodological rigor, and reproducibility of research findings [18]. This approach provides a structured and comprehensive evaluation of existing knowledge on a specific topic, which is particularly important in today’s world, where information grows at an unprecedented pace. The following sections present a detailed explanation of the entire process followed to carry out this review.

This study follows the PRISMA 2020 protocol [19], adapting it flexibly to ensure the integrity and objectivity of the research, while facilitating informed decision-making based on the best available evidence. This method offers a clear and precise framework to outline the applied methodology in the sections that follow. The PRISMA for Abstracts guidelines [20] were also adopted to ensure a structured and transparent reporting of the abstract screening process.

The primary aim of this review is to identify, assess, and synthesize the existing literature on the use of AI algorithms for detecting and predicting hazardous environments and occupational risks in the workplace. Specifically, the review seeks to analyze the methodologies employed, the types of data used, practical applications developed, and the main challenges encountered in the implementation of these solutions.

### 2.1. Mapping Questions

The mapping questions (MQs) used in this study guided both the publication search and the synthesis and classification processes to extract relevant information. The questions are as follows:

- MQ1: How many articles have been published on the application of AI in the detection and prediction of occupational risks in recent years?
- MQ2: Who are the most prolific authors in research on AI algorithms for occupational risk detection?
- MQ3: What types of data are used in studies on AI applications for risk detection?
- MQ4: What specific occupational risk types or scenarios are targeted by AI applications in risk detection and prediction?
- MQ5: Which AI algorithms or techniques are employed to detect and predict occupational risks?

### 2.2. Inclusion and Exclusion Criteria

To carry out an initial selection of the articles, a series of inclusion and exclusion criteria were established, determining whether a publication should be included in the analysis. The entire screening process was performed manually by the authors. However, initial filters for publication date, language, and peer review were applied directly through the search platform functionalities where available, serving as a preliminary sieve. Despite these automatic filters, all retrieved documents were subject to a final, manual screening to ensure that no relevant articles were missed.

- IC1: Accessibility: The publication must be available in an open-access repository or on a platform accessible to the scientific community, ensuring the possibility of peer review by third parties.
- IC2: Language: The publication must be written in English, as it is the predominant language in global scientific literature, ensuring the widest possible dissemination and understanding.
- IC3: Publication date: Only publications from 2019 onward will be considered. Extending the timeframe further would include less relevant papers with limited innovation, while narrowing it might exclude important developments. This range strikes a balance, capturing recent advancements while maintaining a focus on relevant studies.
- IC4: Peer Review: The publication must have undergone peer review, a process that ensures the scientific validity and methodological quality of the studies.
- IC5: Relevance to the Topic: The publication must be directly relevant to the central theme of the study.

The exclusion criteria are derived directly from the inclusion criteria. Any publication that does not meet one or more of the established inclusion criteria will be automatically excluded from the analysis. Additionally, publications consisting of systematic literature reviews were excluded, as this study focuses exclusively on original research and practical applications of AI in the detection and prevention of occupational risks. The type of document was not an exclusion criterion; therefore, journal articles, conference papers, book chapters, and technical reports were all considered as long as they met the established inclusion criteria, due to the emerging and multidisciplinary nature of the topic, where valuable insights often appear outside traditional journals.

### 2.3. Quality Assessment Questions

The purpose of the quality assessment (QA) questions is to conduct a more detailed screening following the initial filtering based on the inclusion and exclusion criteria. The first two questions ensure that the article’s topic is appropriate and addresses the main objectives of the mapping. The remaining four questions focus on the form, structure, and methodological quality of the study, assessing key aspects to guarantee the robustness of the research. For each article, after a detailed reading, the following questions were evaluated:

1. Does the article address the application of AI techniques for the prevention or detection of occupational risks?
2. Does the study present systems with a direct impact on worker safety in workplace environments?
3. Does the article clearly and comprehensively describe the methods used and the results obtained?
4. Does the article position its findings within the context of previous research, discussing its relevance compared to existing knowledge?
5. Are reliable and validated measurement instruments used in the study?
6. Do the article’s findings adequately support the conclusions presented?

Each of these questions is scored according to the clarity and quality of the responses provided by the article. A scoring rubric was applied, assigning values of 0, 0.5, or 1 point per question. The total possible score for each article ranged from 0 to 6 points. To ensure methodological rigor, a minimum threshold was established: studies with a total score of 4.5 or less, or with a score of 0 in any question, were excluded from the analysis. This systematic scoring process ensured that only high-quality studies meeting the established standards were retained for further consideration.

### 2.4. Search Strategy

Four key information sources were used to search for articles: IEEE Digital Library [21], PubMed [22], Scopus [23], and Web of Science [24]. These sources were selected for their high relevance and comprehensive coverage in technology, health, and interdisciplinary research.

The search string was carefully crafted to maximize the inclusion of relevant studies while minimizing the retrieval of irrelevant results, as outlined below.

*(“artificial intelligence” OR “AI” OR “machine learning” OR “ML” OR “deep learning” OR “neural networks” OR “predictive modeling” OR “data-driven models”) AND (“risk prediction” OR “hazard detection” OR “risk assessment” OR “occupational safety” OR “workplace safety” OR “hazard identification”) AND (“detection” OR “forecasting” OR “monitoring” OR “prevention” OR “mitigation” OR “management”) AND (“workers” OR “employees” OR “staff” OR “occupational health”) AND (“worker safety” OR “workplace health” OR “health risks”).*

The Boolean operator OR was used within each conceptual block to capture synonyms and related terms, thereby maximizing sensitivity. The operator AND was applied between blocks to ensure the inclusion of all key concepts simultaneously, thus enhancing specificity and reducing irrelevant retrieval. The combination of OR and AND provides a balanced search: OR broadens coverage within categories, while AND restricts results to their intersection, ensuring both sensitivity and precision in the identification of relevant studies.

In summary, this search strategy follows the standard approach commonly employed in scientific literature, ensuring comprehensive coverage of relevant studies while upholding methodological rigor.

## 3. Review Process

The articles search was conducted on 1 August 2024, resulting in a total of 209 articles, with the source breakdown detailed in Table 1:

A total of 209 articles were collected. The tool Parsifal v2.2.0 [25]—which is particularly suited for such tasks as it facilitates the efficient management of the various stages involved in systematic literature reviews—was used to carry out the screening and selection process.

After the elimination of duplicates, 183 documents remained. A preliminary screening was then conducted based on the titles, abstracts and keywords. During this initial phase, the inclusion and exclusion criteria were applied. This process led to the exclusion of 91 articles for the following reasons: 66 were not relevant to the topic, 16 were systematic literature reviews, 7 were inaccessible, 1 was not in the English language, and 1 had not undergone a peer-review process. After this manual screening, the number of articles was reduced to 92.

These 92 publications underwent the detailed review phase, during which they were fully assessed using the predefined QA evaluation procedure. By applying these criteria, studies that did not reach the established threshold were eliminated, ensuring that only the most rigorous and relevant articles were included in the final analysis.

**Figure 1:**
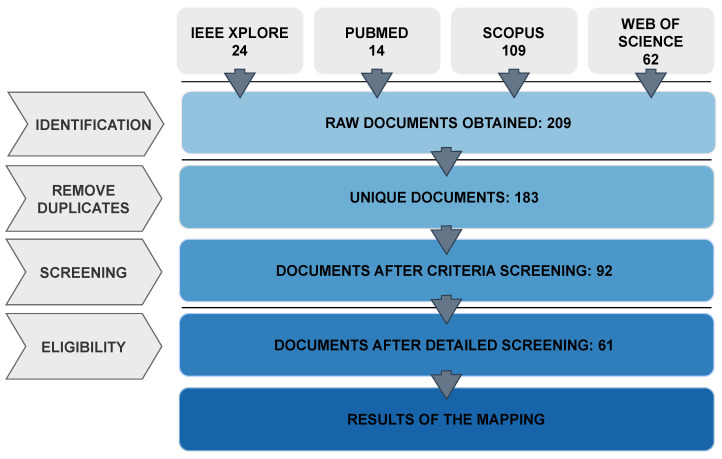
Flow diagram of the review process.

The final outcome of this process was the selection of 61 articles, which formed the basis for answering the mapping questions and conducting the final analysis. The complete list of these 61 articles is provided in the References section, specifically from [26,27,28,29,30,31,32,33,34,35,36,37,38,39,40,41,42,43,44,45,46,47,48,49,50,51,52,53,54,55,56,57,58,59,60,61,62,63,64,65,66,67,68,69,70,71,72,73,74,75,76,77,78,79,80,81,82,83,84,85,86]. Figure 1 presents the flow diagram summarizing the selection process.

## 4. Results

This section presents the main findings obtained from the quantitative and qualitative analysis of the reviewed studies. The quantitative analysis examines the temporal and geographical evolution of publications, identifying the most prolific journals and authors in this field. In contrast, the qualitative analysis explores into the most relevant research topics, the technologies and devices used, as well as the algorithms and frameworks most commonly employed in the studies. This combination of approaches provides a comprehensive view of the trends, challenges, and opportunities in this area of research.

### 4.1. Quantitative Analysis

The quantitative analysis examines the reviewed publications from a numerical perspective, focusing on their temporal evolution, geographical distribution, and authorship patterns. It also identifies the journals with the highest number of contributions to this research field. Together, these indicators provide an objective overview of the development, scope, and dissemination of studies on AI applied to occupational risk prevention.

#### 4.1.1. Distribution of Studies over the Years

**Figure 2:**
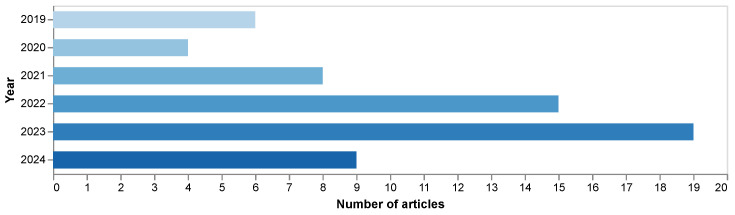
Publications per year (2019–2024).

The number of articles published between 2019 and 2024 shows a clear upward trend, with a peak in 2023, as illustrated in Figure 2. It is important to note that the data for 2024 is preliminary, as the last systematic literature search for this review was conducted on 1 August 2024, covering only the first seven months of the year. During this period, 9 articles were identified for 2024. If this publication rate were to continue consistently for the remaining five months of 2024, the total number of articles for the entire year could potentially reach around 15–16. This projection, while not necessarily exceeding the 2023 peak, indicates a strong, sustained interest in the field, which reinforces the general growth trajectory observed since 2019 and suggests an increasing interest in AI for occupational risk prevention. The dip observed in 2020 can be attributed to the COVID-19 pandemic’s impact on work dynamics.

#### 4.1.2. Geographical Analysis

**Figure 3:**
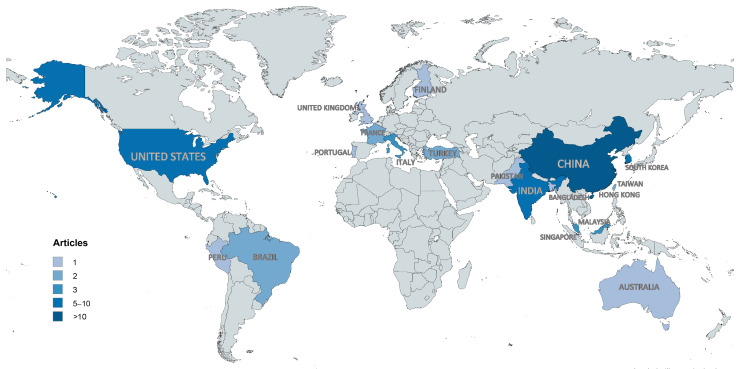
Geographical distribution of studies.

The geographical distribution of publications reveals notable regional patterns in research activity. China leads with 13 publications, followed by South Korea (8), and both India and the United States (7 each). A map of the geographical distribution (Figure 3) highlights contributions from smaller regions such as Hong Kong, Singapore, and Bangladesh.

#### 4.1.3. Most Prolific Authors

**Figure 4:**
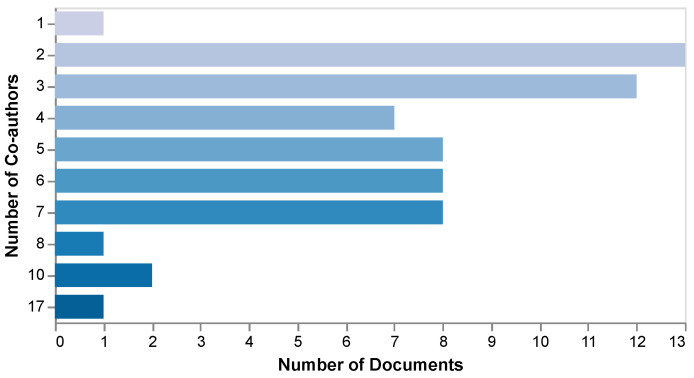
Number of co-authors per article in the reviewed studies.

A total of 265 authors contributed to the reviewed studies. Most articles were written by 2–3 co-authors, though one study included as many as 17, as illustrated in Figure 4. Table 2 lists the most prolific authors, highlighting those who contributed to multiple papers.

#### 4.1.4. Journals with the Highest Number of Publications

Safety Science (8.2%) leads in publications, followed by Automation in Construction (6.56%) and IEEE Access (4.92%). The ‘Others’ category (70.49%) reflects the diversity of journals contributing to AI in occupational safety. To complement this analysis, the reviewed studies for each journal are cited and referenced. The journal impact factor (JIF) values from the Journal Citation Reports (JCRs), the quartile (Q), and their corresponding categories are also provided, as they represent internationally recognized indicators of journal quality and scientific impact (Table 3).

### 4.2. Qualitative Analysis

The qualitative analysis delves deeper into key concepts, areas of application, the technologies employed, and the algorithms used in the reviewed studies. The following is a breakdown of the most relevant elements, providing a detailed overview of current trends in research.

#### 4.2.1. Study Keywords

**Figure 5:**
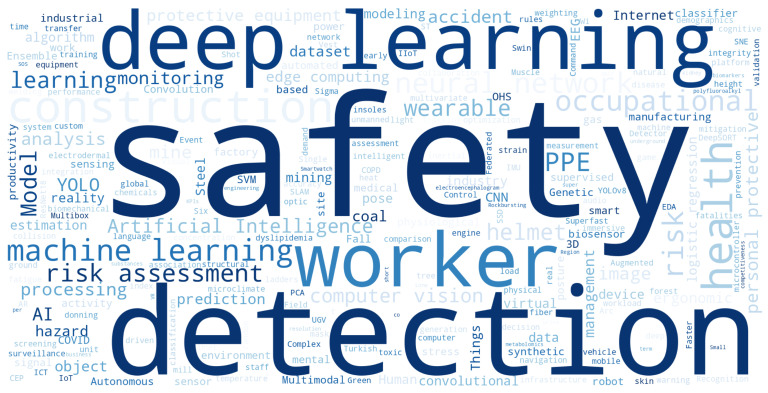
Word cloud of the most relevant keywords in the reviewed studies.

To gain deeper insights into the most researched topics in the field of AI applications for occupational risk detection, a word cloud was generated based on a maximum of 250 keywords, both individual terms and bigrams, as shown in Figure 5. This analysis helps identify the most recurring terms and concepts in the literature.

In the word cloud, the terms detection, safety, and deep learning stand out, highlighting the central objective of the studies: improving safety and reducing accidents in high-risk work environments through the use of advanced AI.

The predominance of techniques such as deep learning and machine learning (ML) clearly indicates a preference for algorithms capable of processing large volumes of data and learning from them. This reflects that research in this field is aligned with current AI trends, prioritizing precision and automation in risk detection.

Terms such as occupational health, construction, and risk assessment suggest a focus on specific sectors, such as construction, and on the evaluation of risks related to occupational health. These concepts reinforce the idea that the studies aim not only to improve efficiency in risk identification but also to protect workers in hazardous industries.

Finally, the presence of terms like PPE and wearable highlights the importance of technological devices and the Internet of Things in the monitoring and control of occupational risks. These terms reflect a comprehensive approach that combines wearable technologies with advanced algorithms to enhance workplace safety.

#### 4.2.2. Main Work Topics and Fields

**Figure 6:**
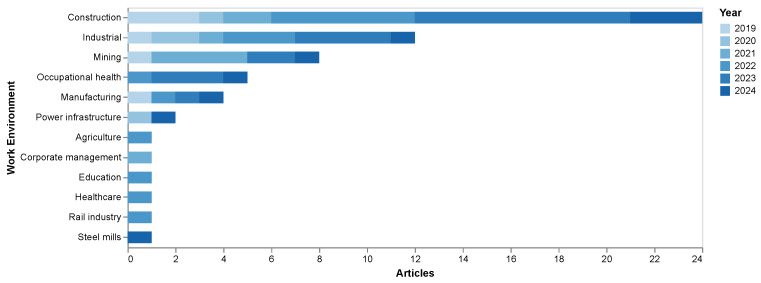
Number of articles by work field, divided by years.

To better visualize the main work topics of the reviewed studies, a graph was generated showing the number of articles associated with each topic, divided by year, as presented in Figure 6. This demonstrates that while certain specific sectors have been less explored, there is a wide range of areas where AI is being applied for the detection and prevention of occupational risks.

Among the topics that clearly stand out in the graph is construction, with the largest number of studies. This was already anticipated in the keyword analysis, and it is unsurprising given that construction is a sector with high occupational risks, which justifies the need for AI-based solutions to improve safety. Other topics with a significant number of publications are industry and mining, both of which are traditionally dangerous sectors, explaining the interest in applying advanced technologies for risk detection in these environments. Manufacturing and occupational health are also prominent, showcasing the versatility of approaches applied to worker protection.

Regarding the years represented by the colors in the graph, there is no clear trend linking specific topics to certain years. The studies are relatively evenly distributed across time in the various sectors, suggesting that there has been no predominant focus on a particular topic in any given year. Research in these fields has been consistent, with no indication that a specific topic has been predominantly explored in any single year.

#### 4.2.3. Devices Used for Data Collection

In the reviewed studies, a total of 10 different types of devices were identified for data collection, all of which are cited and referenced in Table 4. This table provides a comprehensive overview of the most common devices used in the studies and the frequency of their utilization. By consolidating all relevant citations in this table, it ensures transparency and traceability, allowing readers to easily verify the sources and methodologies employed in each study.

Reviewing Table 4, it is clear that cameras are the most widely used device. This predominance may be due to the versatility of cameras, which allow real-time visual data capture. These data can be processed by CV, DL algorithms, such as Convolutional Neural Networks (CNNs), and classic ML algorithms, such as Support Vector Machine (SVM), which are ideal for automatic risk detection in workplace environments.

Wearable sensors are also highly important for data collection, as they enable real-time monitoring of workers, measuring their physiological status and interaction with the environment. These devices are often linked to algorithms for time series analysis and event prediction.

Other notable devices include MEMS sensors and augmented reality (AR) and virtual reality (VR) devices, which allow for detailed monitoring of the environment or the worker in simulated or controlled situations, providing an immersive approach to risk assessment.

Finally, although less common, devices such as smartphones and microphones have been used. Their ability to collect acoustic data or perform basic measurements complements risk detection studies in more specific or low-budget contexts.

#### 4.2.4. Main Artificial Intelligence Algorithms and Tools Used

A total of 109 different AI techniques, algorithms, and tools were identified across all the reviewed articles. Table 5 shows the techniques that have been used in at least two different studies, including both algorithms and frameworks or libraries that facilitate the development of AI-based solutions.

As shown in Table 5, CV is the most widely used technique in these studies, with YOLO and CNN being the most employed algorithms. This makes sense, as the previous section noted that cameras were the most commonly used device, reinforcing the choice of these algorithms. The use of frameworks also stands out, with TensorFlow being the most commonly used among the studies.

Although many works utilize more modern AI techniques, there is also a notable presence of classic ML algorithms, such as SVM and Random Forest. This is interesting, given that all reviewed articles are from 2019 onwards, indicating that these techniques remain useful when combined with more advanced methods.

## 5. Detailed Analysis

**Figure 7:**
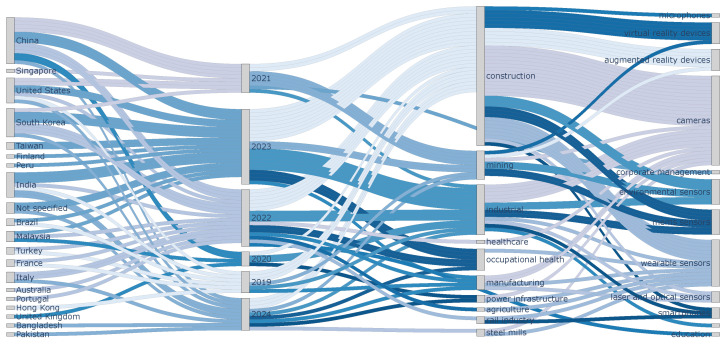
Sankey diagram illustrating the relationships between countries, years, main work topics, and devices. The color-coding system is designed such that all incoming connections to each node share the same color.

To provide a more comprehensive view of the trends identified in the reviewed studies, a Sankey diagram was generated, as shown in Figure 7, visualizing the relationships between countries, years, main work topics, and devices used. In this diagram, the links are color-coded such that all incoming connections to a node share the same color, creating a clear contrast between the categories and facilitating the identification of patterns and trends.

The visual representation in the Sankey diagram serves as a powerful tool to understand the flow and evolution of research in this domain, connecting the origins of the studies with their methodological and thematic focus. In the following subsections, we will provide a detailed analysis of the key relationships depicted in the diagram, focusing on the connections between countries and years, years and main topics, and the main topics and devices employed.

### 5.1. Relationship Between Countries and Years

Several interesting patterns can be identified in the evolution of studies over time upon examining the relationship between countries and years in the Sankey diagram. In 2019, research on AI for occupational risk detection was fairly dispersed, with each country represented by only one study.

In 2020, nearly all efforts were led by the United Kingdom. This predominance may be linked to the global COVID-19 pandemic, which restricted in-person studies in many other countries. This situation may have forced researchers in other regions to pause their projects or shift priorities, while studies continued in the United Kingdom.

The situation changed in 2021 and 2022, where a wider variety of countries were observed to be involved in the research, indicating that AI and occupational risk research began to gain global traction, possibly due to the resumption of in-person activities and the emergence of new technologies and tools to carry out studies in this field.

In 2023 and 2024, the participation of countries such as China, South Korea, and India intensified, showing an increase in the number of published articles. This highlights Asia’s growing role as a leader in technological research applied to workplace safety. Furthermore, in the last year, there has been greater diversity in the countries participating in these studies, including nations such as Bangladesh and Pakistan, indicating that this research is reaching developing countries and regions where there was previously less presence.

### 5.2. Relationship Between Years and Main Topics

Analyzing the relationship between the years and the main topics of the studies in the diagram, no clear trend towards a specific topic in a particular year is observed, as seen previously in Table 4. Even in 2020, despite the global interruption caused by the pandemic, studies continued on topics such as construction, power infrastructure, and steel mills, indicating that even during the most challenging years, research in high-risk sectors did not completely halt. This highlights the importance of occupational safety, regardless of external circumstances.

### 5.3. Relationship Between Main Topics and Devices Used

While not much information can be drawn for certain topics due to the presence of only one study, such as corporate management, rail industry, or education, clear trends can be identified for construction and industrial topics, which warrant deeper analysis.

#### 5.3.1. Construction

In the case of construction, cameras are the most common device used in the majority of studies, with a clear methodology: capturing images and videos that are then processed using advanced DL algorithms such as YOLO and CNNs. Several papers have leveraged this technology for detecting the use of PPE and monitoring safety on construction sites. For example, studies such as [56,57] implement versions of YOLOv5 and ResNet to detect safety helmets and verify whether workers are wearing the required equipment. This approach is quite common, as PPE detection is crucial to prevent accidents in high-risk environments such as construction.

Other studies, such as [43], go beyond PPE detection by utilizing YOLOv3 and LSTM to analyze images and predict worker falls. This type of analysis is particularly relevant in construction sites, where the risk of falls is one of the primary concerns. Similarly, the use of YOLOv4-Tiny in combination with Deep SORT and the Kalman filter in [32] allows for real-time visual warnings through mixed reality technologies, enhancing the ability to respond to hazardous situations.

VR and AR devices, in combination with conventional cameras, play a significant role in simulating dangerous work environments. In the work by [45], a game engine is employed to generate synthetic data and improve the training of models such as YOLOv5, allowing workers to practice in simulated scenarios without exposing themselves to real risks. These approaches enable the combination of detection algorithms with digitally generated 3D environments, which is particularly useful for safety training.

Although PPE detection and fall prevention are the most common themes, some studies go further by focusing on ergonomic assessment and real-time workload analysis. The study conducted by [54] uses Faster R-CNN to analyze workers’ postures and assess musculoskeletal risks, while in [42], portable sensors and cameras are combined to estimate the biomechanical load on employees. These studies introduce a more holistic approach to safety analysis, using technologies that allow for the monitoring of both physical risks and ergonomic conditions for workers.

More specialized studies are also observed, such as [51], where an autonomous robot equipped with YOLOv3 and SLAM technology is used to detect workers not wearing PPE, highlighting the integration of AI into robotic solutions for occupational safety. These innovations not only identify risks but also enable real-time actions using advanced perception and autonomous navigation algorithms.

#### 5.3.2. Industry

In the industrial sector, cameras also play a key role in detecting PPE and improving workplace safety. Similar to the construction field, the use of YOLO and other DL algorithms is common. For instance, in studies such as [53], a combination of YOLO, R-CNN, and CNN is used to detect a variety of safety equipment in real time, such as helmets, gloves, vests, and goggles, by analyzing video sequences.

A more innovative approach is presented in [58], which combines OpenCV, TensorFlow, and SSD MobileNet to detect and prevent accidents in industrial shredding machines. This study stands out for its preventive approach, using cameras to detect the proximity of human hands to the machine and triggering safety mechanisms before an accident occurs. This type of solution is particularly useful in industrial environments where accidents involving heavy machinery are frequent.

It is also noteworthy to observe the trend towards using lightweight and high-speed models in industrial settings. For example, in [38], a combination of YOLOv5, CSPNet, and PANet is used, along with optimization techniques such as Network Slimming and Channel Pruning, to create a PPE detector that operates at over 100 fps. This model is employed in both cameras and smartphones, allowing for flexible and fast application in industrial environments where detection speed can prevent accidents.

However, not all solutions rely exclusively on cameras. MEMS sensors also play an important role in monitoring safety in industrial settings. In the study [68], MEMS sensors and Logistic Regression are used to determine whether a helmet is being worn by analyzing microclimate data around the worker. This approach is particularly useful in situations where cameras are insufficient, such as in enclosed spaces or where visual conditions are not optimal.

Another interesting example is the use of environmental and MEMS sensors in welding. The study by [69] implements an autonomous AI-controlled welding system that uses Raspberry Pi and sensors to assess risks in real time during the welding process. This approach not only detects risks but also automatically adjusts welding parameters to minimize hazards, representing an advanced integration of AI into industrial machinery for accident prevention.

#### 5.3.3. Mining

In the mining sector, MEMS sensors and environmental sensors are responsible for real-time risk monitoring due to the hazardous conditions that prevail in underground environments. Unlike sectors such as construction or industry, where cameras dominate for visual risk detection, mining requires solutions capable of operating in low-visibility conditions and managing multiple critical environmental variables.

For example, in the study conducted by [39], a monitoring and alert system is implemented that combines MEMS and environmental sensors with CNN and MobileNet to detect structural hazards and the accumulation of toxic gases in coal mines. Another interesting approach is presented in [70], where an advanced AR system is used in combination with data from MEMS and environmental sensors. This study stands out by employing Deep Boltzmann Machines and Gaussian RBF kernel SVM to process multimodal data in real time, enabling the prediction of emergency situations before they occur. Additionally, the AR system provides workers with immersive visualizations that enhance decision-making in high-risk scenarios, offering real-time visual warnings and facilitating the management of critical situations through Command and Control Systems.

#### 5.3.4. Other Sectors

In addition to the most-studied sectors such as construction, industry, and mining, AI applications have also been explored in occupational health, steel mills, electrical infrastructure, and agriculture, although to a lesser extent. In the area of occupational health, remote monitoring systems based on wearable sensors are noteworthy. For example, the research conducted by [64] shows that algorithms such as SVM, Linear Discriminant Analysis, and Artificial Neural Networks are combined to detect anomalies in workers’ health and risks related to COVID-19. The use of these sensors enables continuous and real-time monitoring of employee health, optimizing responses to potential risks.

In steel mills, safety is managed through a combination of wearable sensors and cameras. As shown in [49], DL algorithms like YOLO and SVM are used to detect the use of PPE and unsafe conditions. This approach enhances safety in highly hazardous work environments such as steel mills, where occupational risks are elevated due to extreme working conditions.

For electrical infrastructure, AI ensemble models are used to manage worker safety. In [52], an autonomous platform is implemented that combines models like YOLOv5, TadGAN, and ensemble techniques such as Bagging and Boosting, alongside a large-scale data processing ecosystem based on technologies like Hadoop and Spark, to provide real-time safety monitoring and alerts. This combination of techniques allows for effective safety management in large electrical infrastructures, where the risk of accidents is high.

Lastly, in the field of agriculture, research has focused on mitigating the risk of musculoskeletal disorders in agricultural operators through the use of wearable sensors and vibration data analysis. The study conducted by [65] employs KNN algorithms to map and assess vibration exposure in operators, contributing to the prevention of posture-related and repetitive motion disorders in agricultural environments. This approach is notable for its ability to identify ergonomic risks through Agriculture 4.0 technology.

## 6. Discussion

The analysis of the reviewed studies on the application of AI algorithms for occupational risk detection highlights several important trends and challenges in this rapidly evolving field. Since 2019, there has been a marked increase in the volume of published research, particularly from 2021 to 2023, despite a temporary dip in 2020 likely due to the COVID-19 pandemic. This growing interest reflects a rising awareness of the importance of worker safety and the potential of AI technologies to address longstanding challenges in occupational risk management. The data reveal that AI applications are becoming more diverse and advanced, with an increasing number of studies exploring AI’s role in risk detection across a range of industries.

An additional strength of this study lies in the consistency between the search strategy and the results obtained. Many of the terms that emerged as central in the word cloud, such as detection, deep learning, machine learning, and risk assessment, directly overlap with those employed in the initial search string. This overlap reinforces the robustness of the methodological approach, confirming that the retrieved literature accurately reflects the targeted key concepts and research directions. The alignment between predefined search terms and emerging themes also validates the inclusion criteria and supports the representativeness of the studies analyzed.

The first key observation is the increasing number of publications in recent years. A significant surge in research on AI-driven safety solutions can be seen starting in 2021, peaking in 2023. Preliminary data for 2024 suggests a strong, sustained interest, indicating the field continues its upward trajectory as industries and researchers seek ways to address workplace safety more effectively. The focus on AI in occupational risk detection aligns with broader global trends that emphasize worker protection and the integration of digital technologies in various sectors. By 2023, over 100 articles were published in this domain, with substantial contributions from countries such as China, South Korea, India, and the United Kingdom. This indicates a strong international commitment to applying AI for improving workplace safety, particularly in high-risk sectors like construction, manufacturing, and mining.

In terms of the specific types of data used, visual data, especially from cameras, has been the most prevalent. More than 40% of the studies reviewed rely on visual data to monitor worker behavior, PPE usage, and potential hazards in real time. Algorithms like YOLO and CNN are frequently employed to process this data for detecting unsafe actions or environmental risks. Visual data is particularly useful in environments where immediate, real-time analysis is required, such as construction sites where monitoring worker behavior or equipment operation can prevent accidents. However, visual data also presents challenges, especially in low-visibility environments, such as underground mining or poorly lit factory floors. As a result, many studies have started integrating additional data from wearable sensors, MEMS, and environmental sensors to supplement the visual data and provide more robust, real-time monitoring. This multimodal approach allows for a more comprehensive understanding of the worker’s environment and physical state, improving the accuracy of risk predictions.

The focus of AI applications in occupational risk detection has largely been on high-risk sectors, particularly construction, mining, and manufacturing, where workers face significant physical dangers. In construction, the most common risks addressed by AI applications are falls, accidents related to heavy machinery, and PPE compliance. AI algorithms are often used to detect whether workers are wearing the required protective equipment or are working in hazardous environments. In mining, the challenge lies in monitoring air quality and detecting structural instability in underground environments. AI-based solutions that integrate sensor data to monitor gas levels, vibrations, and air quality have shown promise in mitigating these risks. In manufacturing, AI solutions are typically employed to prevent repetitive strain injuries and other musculoskeletal disorders by tracking worker movements and monitoring ergonomics. Additionally, the healthcare and agricultural sectors are starting to explore AI applications, with a focus on monitoring worker health and preventing physical strain.

The use of AI algorithms in these contexts predominantly involves DL techniques. CNN, particularly in object detection tasks, and YOLO models for real-time detection, are the most commonly used methods. These algorithms excel at processing large amounts of visual data, making them particularly suited for monitoring worker safety in environments where real-time decisions are critical. Despite their effectiveness, the reliance on DL models presents challenges, particularly in terms of transparency and interpretability. This is especially crucial in high-stakes environments where safety decisions based on AI predictions can directly impact workers’ lives. Additionally, while DL models have proven effective in controlled settings, their application in cluttered, poorly lit, or dynamically changing environments, such as mining tunnels, is still a significant challenge. The ability to adapt these models to various operational contexts remains a key area of research.

While DL has dominated the field, traditional ML algorithms such as SVM, Random Forests, and KNN still play an important role, especially in the analysis of sensor data. These models are frequently used in conjunction with wearable sensors to predict risks related to worker health, such as fatigue or exposure to harmful substances. The combination of these classical models with modern DL techniques reflects an ongoing trend towards hybrid approaches, where the strengths of both types of algorithms are leveraged to create more accurate and adaptable systems for occupational risk detection.

Despite the significant progress made in this field, several challenges remain. One of the most pressing concerns is the over-reliance on visual data, which is limited in environments where visibility is poor, such as in underground mines or in factories with low lighting. In these contexts, the performance of camera-based systems is frequently affected by insufficient illumination, airborne particles, or structural obstacles, which compromise their reliability and accuracy. Emerging alternatives such as LiDAR, radar-based motion sensing, edge-level sensor fusion, and fiber-optic sensing are being explored as solutions. These technologies can provide more resilient and precise monitoring in adverse conditions, particularly in scenarios characterized by low light, dust, or obstructions, where traditional vision systems lose effectiveness. Although some studies have incorporated supplementary data from sensors to address this limitation, the integration of multimodal data (visual, environmental, and physiological) still faces technical and methodological challenges. Envisioned multimodal inputs encompass audio recordings, thermal images, inertial measurements from wearable devices, data from environmental sensors (e.g., gas levels, temperature, air quality), and biometric indicators. Harnessing such heterogeneous sources demands addressing several challenges, including the synchronization and preprocessing of diverse data streams, the temporal and spatial alignment of signals, and the development of models capable of jointly integrating and learning from multiple modalities. More research is needed to develop integrated AI systems that can effectively combine these different data streams to provide more comprehensive, context-aware risk detection.

Another challenge is the lack of standardized methodologies for implementing AI-based risk detection systems. Given the diversity of work environments and the wide range of risks involved, it is difficult to apply a single approach universally across all industries. This fragmentation in methodologies makes it difficult to compare studies or replicate findings across different settings. The development of standardized frameworks and protocols for the implementation of AI in occupational safety would help overcome this issue and facilitate the broader adoption of AI technologies.

Finally, the issue of explainability in AI models remains a key concern. In safety-critical environments, it is crucial for AI systems to not only make accurate predictions but also provide transparent explanations for their decisions. This is especially important in industries where safety regulations require human oversight and accountability. The development of explainable AI techniques is essential to ensure that AI systems are trusted by workers, supervisors, and regulatory bodies, and to facilitate the responsible integration of AI in occupational safety.

Beyond these technical and methodological challenges, it is also crucial to address the long-term societal implications of widespread AI adoption in occupational safety. These include potential worker displacement in roles heavily reliant on manual supervision. Privacy concerns also arise with constant monitoring through sensors and cameras. Addressing these issues requires transparent data policies, stakeholder involvement in system design, and strong regulatory frameworks to protect worker rights and foster trust.

## 7. Future Work

This paper has presented a comprehensive review of the current state of AI applications in occupational risk detection, highlighting trends, methodologies, and challenges. While it has successfully provided an in-depth analysis, there are several areas where future work could build upon these findings.

One promising future direction is the development of multimodal approaches that more effectively integrate visual, sensory, and environmental data. By combining camera-based systems, wearable sensors, and environmental monitors, it is possible to create more accurate, real-time risk detection systems, particularly in high-risk industries such as mining, construction, and manufacturing. These multimodal systems could address the current limitations of visual data alone, especially in environments with poor lighting or complex physical conditions. Future research should focus on improving the fusion of these data streams and refining machine learning algorithms to process and analyze this integrated data effectively. This would not only enhance the accuracy of risk prediction but also offer more adaptive solutions tailored to diverse workplace environments.

To further advance the field of AI in occupational safety, promoting standardization and fostering international collaboration are paramount. This involves establishing sector-specific working groups dedicated to the development of open datasets, benchmarking protocols, and comprehensive evaluation frameworks tailored to occupational safety applications. Crucially, collaboration between academia, industry stakeholders, and regulatory bodies will be essential for defining robust technical standards and ethical guidelines that ensure the responsible and effective deployment of AI solutions across diverse work environments.

Another pivotal area for future investigation is the establishment of robust frameworks for standardization and enhanced collaboration within the occupational safety domain. Currently, the diverse range of work environments and inherent risks often leads to a fragmentation in methodologies, complicating the comparison of studies and the replication of findings across different settings. To overcome this challenge, it is imperative to foster the creation of sector-specific working groups. These collaborative entities, involving academia, industry stakeholders, and regulatory bodies, would play a crucial role in developing essential resources. Specifically, their efforts should focus on defining open datasets, common benchmarking protocols, and comprehensive evaluation frameworks tailored to the unique demands of occupational safety applications.

Furthermore, standardization should extend to several key technical areas to ensure the consistent and reliable deployment of AI solutions. This includes defining uniform data collection protocols, such as precise guidelines for sensor calibration and positioning to ensure data consistency. It also necessitates the development of standardized annotation schemes for effectively classifying and labeling various risk scenarios and hazardous behaviors. Moreover, future work must focus on developing rigorous model validation procedures that account for diverse real-world contexts and operational environments, moving beyond idealized laboratory settings. Crucially, the evaluation of AI systems in occupational safety needs to broaden its scope beyond mere accuracy, incorporating more comprehensive performance metrics that reflect practical considerations like reliability, real-time responsiveness (latency), and the interpretability of model outputs in real-world deployments. These collective efforts are fundamental to facilitating the broader adoption of trustworthy and effective AI technologies in workplace safety.

Another critical area for future research is the adoption and rigorous exploration of interpretable AI (XAI) techniques. While DL models have demonstrated considerable accuracy in occupational risk detection, their inherent lack of transparency often presents a significant impediment to widespread adoption in industrial settings where accountability and trust are paramount. Making system decisions comprehensible is crucial for their integration.

To address this challenge, several promising XAI approaches for occupational safety include attention mechanisms, saliency maps, decision trees integrated with DL models, and counterfactual explanations. These techniques are not merely about revealing a model’s ’thinking’; they empower users to understand *why* a particular risk prediction was made, enabling workers and supervisors to discern the underlying rationale of system decisions. For instance, counterfactual explanations can illustrate what minimal changes in input data would have altered a prediction, providing actionable insights. By integrating these XAI methods, researchers can enhance the interpretability of models, thereby fostering greater acceptance among crucial stakeholders such as workers, safety officers, and regulatory bodies. Ultimately, improving the transparency of AI systems is fundamental to ensuring their responsible, ethical, and effective deployment in safety-critical applications.

In addition to these advancements, the integration of advanced wearable technologies, including biometric sensors and AR devices, presents another exciting avenue for future research. These technologies are still in the early stages of exploration compared to more established camera-based systems but hold great potential. Biometric sensors, for example, could monitor worker health metrics such as heart rate, fatigue levels, or exposure to hazardous substances, while AR devices could provide real-time safety alerts or visual guidance. The combination of these technologies with ML algorithms could offer a more comprehensive and personalized risk assessment, improving both individual worker safety and overall workplace conditions.

Furthermore, and beyond the high-risk sectors traditionally addressed in the study of AI for occupational risk prevention (such as construction, mining, and manufacturing), an area of future research of great relevance is the expansion of AI applications to a wider range of industries. There is significant potential in sectors such as agriculture, maritime operations, the oil and gas industry, logistics, and emergency response—among others. These industries are characterized by complex and dynamic environments, which often have high incident rates. Therefore, future research should focus on developing and implementing advanced safety monitoring and prediction systems specifically designed to address the challenges inherent in these contexts, which represents a promising line of work for improving occupational safety globally.

Additionally, to ensure the global relevance and impact of AI in occupational safety, future efforts should actively focus on increasing inclusivity in research. This involves supporting open-access publishing, incentivizing cross-border collaborations, and providing targeted funding for underrepresented regions. Encouraging multilingual dissemination and engaging with local institutions and governments are also crucial strategies to bridge regional research gaps and foster a more equitable and comprehensive body of knowledge in this vital field.

While significant progress has been made in the application of AI for occupational risk detection, several limitations of this study must be acknowledged. The analysis presented here is based on a review of existing literature, which may have inherent biases due to the selective nature of the studies published. Not all industries or geographic regions may be equally represented in the research, potentially limiting the generalizability of the findings. Moreover, while this review provides a comprehensive analysis up to 1 August 2024, the rapidly evolving nature of AI in occupational safety means new developments may have emerged since the time of the review. To maintain its long-term relevance, a follow-up study of this review is planned every two to three years. This would involve updating the literature search to include recent publications, particularly from emerging domains such as generative models or newer sensing technologies. The updated analysis would also examine how newly proposed algorithms and tools are being integrated into practical occupational safety applications, alongside more comprehensive longitudinal studies that track the long-term impact of AI applications on occupational safety outcomes and worker health.

A promising direction for future research involves extending the methodology applied in this review to fire safety, specifically in evacuation modeling and risk prediction in built environments during fire scenarios. This could include a comprehensive analysis of how advanced sensing and modeling techniques are being used to enhance building safety, optimize evacuation strategies, and support real-time decision-making during fire events. The integration of AI systems into fire safety represents a field with significant potential for prevention and response, complementing existing AI applications in occupational safety, along with the analysis of the real development of the fire and its consequences on buildings [87].

This paper has highlighted the substantial progress made in applying AI to occupational risk detection and prediction. The insights gained from the review provide a solid foundation for further exploration and development in this area. However, the field is still in its early stages, and numerous challenges remain. Future work should focus on refining multimodal data integration, enhancing the transparency of AI models, and exploring the potential of emerging wearable technologies. While AI applications in occupational risk detection have already demonstrated their potential, continued innovation and research will be essential to unlock their full promise and ensure safer workplaces for all. The findings of this paper contribute to this ongoing effort and serve as a stepping stone for future advancements in the field.

## Figures and Tables

**Table 1:** Number of articles per platform.

Articles	Platform
24	IEEE Digital Library
14	PubMed
109	Scopus
62	Web of Science

**Table 2:** Most prolific authors.

Art.	Authors
3	Houtan Jebelli, Chansik Park
2	Yizhi Liu, Dongmin Lee, Sharmila G, Jing Li, Jie Wang,
	Jongpil Jeong, Jianhui Wu, Rabia Khalid, Sharjeel Anjum,
	SangHyun Lee, Amit Ojha

**Table 3:** Top journals on AI in occupational risk detection, including their JCR metrics (2024).

Journal	Art.	Studies	Percent	JIF	Q	Category
Safety Science	5	[26,27,28,29,30]	8.2%	4.7	Q1	Engineering, Industrial; Operations Research and Management Science
Automation in Construction	4	[31,32,33,34]	6.56%	9.6	Q1	Construction and Building Technology; Engineering, Civil
IEEE Access	3	[35,36,37]	4.92%	3.4	Q2	Computer Science, Information Systems; Engineering, Electrical and Electronic; Telecommunications
Others	43	–	70.49%	–	–	–

**Table 4:** Devices used for data collection and their frequency in studies.

Device	Number of Studies	Studies
Cameras	23	[32,36,38,39,40,41,42,43,44,45,46,47,48,49,50,51,52,53,54,55,56,57,58]
Wearable sensors	12	[27,29,42,49,52,59,60,61,62,63,64,65]
Environmental sensors, microphones	8	[34,39,48,66,67,68,69,70]
MEMS sensors	7	[34,39,48,66,68,69,70]
Virtual reality devices, augmented reality devices	6	[29,32,43,45,62,70]
Smartphones, laser and optical sensors	6	[31,38,51,59,63,71]
Not specified	21	[26,28,30,33,35,37,72,73,74,75,76,77,78,79,80,81,82,83,84,85,86]

**Table 5:** Most frequently used AI algorithms, frameworks, and tools in the reviewed studies. Acronyms: KNN = K-Nearest Neighbors; LSTM = Long Short-Term Memory; ANN = Artificial Neural Network; BiLSTM = Bidirectional Long Short-Term Memory; ReLU = Rectified Linear Unit; XGBoost = Extreme Gradient Boosting; MLP = Multilayer Perceptron; LDA = Linear Discriminant Analysis; DNN = Deep Neural Network; R-CNN = Region-Based Convolutional Neural Network; PANet = Path Aggregation Network.

Technique or Tool	Art.
YOLO	17
CNN	16
SVM	11
Random Forest, Logistic Regression	7
KNN	5
ResNet, Naive Bayes, Decision Tree, LSTM	4
SSD-MobileNetV2, TensorFlow, ANN	3
TensorFlow Lite, BiLSTM, BP Neural Network, Genetic Algorithm, ReLU, XGBoost, MLP, LDA, Deep SORT, DNN, Neural Network, Faster R-CNN, PANet	2

## Data Availability

This systematic review is based entirely on publicly available literature. All data analyzed in this study are derived from the 61 research articles identified through the systematic search process, which are fully cited in the reference list.
